# Physiological and Pathological Impact of Blood Sampling by Retro-Bulbar Sinus Puncture and Facial Vein Phlebotomy in Laboratory Mice

**DOI:** 10.1371/journal.pone.0113225

**Published:** 2014-11-26

**Authors:** Anne Charlotte Teilmann, Andreas Nygaard Madsen, Birgitte Holst, Jann Hau, Björn Rozell, Klas Stig Peter Abelson

**Affiliations:** 1 Department of Experimental Medicine, Faculty of Health and Medical Sciences, University of Copenhagen, Copenhagen, Denmark; 2 The Novo Nordisk Foundation Center for Basic Metabolic Research, University of Copenhagen, Copenhagen, Denmark; USGS National Wildlife Health Center, United States of America

## Abstract

Retro-bulbar sinus puncture and facial vein phlebotomy are two widely used methods for blood sampling in laboratory mice. However, the animal welfare implications associated with these techniques are currently debated, and the possible physiological and pathological implications of blood sampling using these methods have been sparsely investigated. Therefore, this study was conducted to assess and compare the impacts of blood sampling by retro-bulbar sinus puncture and facial vein phlebotomy. Blood was obtained from either the retro-bulbar sinus or the facial vein from male C57BL/6J mice at two time points, and the samples were analyzed for plasma corticosterone. Body weights were measured at the day of blood sampling and the day after blood sampling, and the food consumption was recorded automatically during the 24 hours post-procedure. At the end of study, cheeks and orbital regions were collected for histopathological analysis to assess the degree of tissue trauma. Mice subjected to facial vein phlebotomy had significantly elevated plasma corticosterone levels at both time points in contrast to mice subjected to retro-bulbar sinus puncture, which did not. Both groups of sampled mice lost weight following blood sampling, but the body weight loss was higher in mice subjected to facial vein phlebotomy. The food consumption was not significantly different between the two groups. At gross necropsy, subcutaneous hematomas were found in both groups and the histopathological analyses revealed extensive tissue trauma after both facial vein phlebotomy and retro-bulbar sinus puncture. This study demonstrates that both blood sampling methods have a considerable impact on the animals' physiological condition, which should be considered whenever blood samples are obtained.

## Introduction

Blood sampling in laboratory mice is a routine experimental procedure. Ideally, blood sampling should be minimally invasive and have minimal impact on both the wellbeing of the animals [1]–[3] and the experimental data [4], [5].

Retro-bulbar sinus puncture (RSP), often also referred to as retro-orbital sinus puncture [5], [6], in laboratory rodents is a commonly used technique for blood sampling. The method, however, is controversial and debated [7]. While some authors claim that an experienced operator will not inflict serious harm to the animals [8], [9], others criticize the method for its potential of seriously damaging the orbital tissues [10]–[12] as well as impairing the animals' wellbeing [13], [14]. Most of these studies, however, have been performed on rats, and an assessment of the method in mice is highly motivated.

Another frequently used blood sampling method in mice is facial vein phlebotomy (FVP) or (sometimes) puncture of the superficial temporal vein [15]. Despite its widespread use few studies have investigated the potential impact of this technique in mice [16]. The method has the potential of damaging the inner ear as well as the major masticatory muscles, thereby causing significant stress and pain as well as functional impairment of mastication. Furthermore, this technique has been associated with a low rate of uncontrollable hemorrhage [15], [16] and thus the mice may be at risk of hypovolemic shock and death.

FVP and RSP are both methods that allow sampling of larger (0.2 – 0.5 ml) blood volumes [17], [18], as opposed to sampling from the tail veins, which normally yields volumes of 0.1 – 0.15 ml [11]. For this reason, FVP and RSP are widely used for routine blood sampling in mice, although in survival studies less blood may be obtained according to general guidelines [11], [19]. To our knowledge, no studies have compared the physiological and tissue level effects of blood sampling using these two techniques in mice. As RSP has been widely criticized, FVP is often referred to as a better method for blood sampling, but without a proper evaluation such claims will lack scientific foundation.

The aim of the present study was to compare stress levels in mice by measuring corticosterone levels, body weight and food consumption during 24 hours after blood sampling with either RSP or FVP. Furthermore, the degree of tissue trauma related to the two procedures was studied by histopathology. The hypothesis was that FVP is not a less stressful method for blood sampling in mice compared to RSP. It was expected that mice subjected to FVP would express equal or higher levels of stress in relation to blood sampling than mice subjected to RSP. The degree of tissue trauma was expected to reflect the invasiveness of the method, with local acute inflammatory reactions as sequelae in both groups.

## Materials and Methods

The experiment was approved by The Animal Experiments Inspectorate under the Danish Ministry of Food, Agriculture and Fisheries (license number: 2014-15-2934-01055). The mice were handled by experienced personnel at all times, in compliance with the *Guide for the Care and Use of Laboratory Animals* [20] in a fully AAALAC accredited facility. Routine health monitoring was based on the FELASA guidelines [21]. The animals had not been tested positive for any of the pathogens on the FELASA list.

In total, 32 five months old male C57BL/6J mice, weighing 30.5±2.3 g (mean ± SD), were randomly divided into three groups: One control group (Group 1, N = 8), one group subjected to FVP (Group 2, N = 12) and one group subjected to RSP (Group 3, N = 12). The mice had previously been used in another study at the same facility, but in compliance with the three Rs in terms of reduction [22], the mice were re-used for this purpose. The mice were allowed a three-month acclimatization period prior to the present study. The mice had not previously been subjected to blood sampling with any of the two techniques used in the present study.

### Housing

The mice were housed in groups of four in a feeding monitoring system (HM-2, MBRose, Faaborg, Denmark). In relation to the present study, they were numbered and the numbers were then randomly distributed between the groups, such that all cages contained mice from all three groups without physically mixing the animals. This was done to avoid cage associated bias in relation to the blood sampling and to avoid intermale aggression in relation to the establishment of new groups. The mice had been chipped (Datamars, Wobum, USA) inter-scapularly at seven weeks of age, in connection to the previous study, which was reused in the present study for individual recognition in the feeding system.

The individual mice were recognized by the system as they entered a feeding hub. Two feeding hubs were available in each cage to avoid aggression in relation to feeding. The food consumption was recorded automatically during four days prior to experimentation and during the 24 hours after blood sampling. To be able to sample blood at peak and nadir within laboratory working hours, artificial lights were switched on from 11 pm, thereby maintaining a reversed diurnal rhythm with a 12∶12 hour light-dark cycle. This was done from two weeks prior to experimentation to allow sufficient habituation to the photoperiod shift [23], [24]. Cage temperature was kept at 22°C±2°C, relative humidity between 45–65%, and the air was exchanged 75 h^−1^. Wooden chips (Tapvei Oy., Kortteinen, Finland) were used as bedding material. Bite bricks (Tapvet, Kortteinen, Finland), Enviro-dri nesting materials (Shepherd Specialty papers, Quakertown, Pennsylvania, USA) and cardboard houses (Brogaarden, Gentofte, Denmark) were used for environmental enrichment. At Day 1, all mice were weighed in connection to the first blood sample, but after the sample had been obtained to avoid plasma corticosterone being influenced by the weighing. Control mice were, however, weighed immediately prior to decapitation. Weighing, decapitation and blood sampling were performed within 60 seconds from the mouse had been removed from the cage. As plasma corticosterone increases in the circulation within 2 – 3 minutes after a stressful stimulus [25]–[27], the blood samples from the mice in Group 1 served as controls for the samples taken by FVP and RSP. At Day 2, the mice in Groups 2 and 3 were weighed in the morning prior to euthanasia and necropsy.

### Blood sampling

The mice of Group 1 were decapitated and trunk blood was collected to obtain levels of plasma corticosterone unaffected by stress. Four mice were euthanized at 9 am, corresponding to 4 pm in the reversed light cycle, and four mice were euthanized at 11 am, corresponding to 6 pm in the reversed light cycle. The corresponding reversed time points are from this point used throughout the manuscript.

The mice of Group 2 were restrained by a firm grip of the scruff and the facial vein was punctured at the lateral side of the cheek at 4 and 6 pm using a 5 mm Goldenrod Lancet (MEDIpoint, New York, USA). The right facial vein was punctured for the first blood sample and the left facial vein for the second blood sample. To quantify the exact sample volume, a 75 µl EDTA coated capillary tube (Vitrex Medical, Herlev, Denmark) was applied to the puncture site and the blood sample was collected in the capillary tube. To avoid damage to the inner ear, the experimenter aimed at puncturing the facial vein and not the superficial temporal vein [28], [29].

The mice of Group 3 were restrained as the mice in Group 2, and blood was sampled at 4 and 6 pm by puncture of the retro-bulbar sinus from the medial canthus of the eye using clean 75 µl EDTA coated capillary tubes [30]. The blood was collected from the right sinus for the first blood sample and from the left sinus for the second blood sample.

After the blood samplings, the stasis (restraint) was released and the mice were returned to their cages. Blood samples were taken by skilled personnel using the techniques on a routine basis. One researcher, who had experience in FVP, sampled the mice of Group 2 and another researcher, experienced in RSP, sampled the mice of Group 3. No anesthesia was applied in connection to the blood sampling to minimize unequal variation between animals. All blood samples were obtained between ±15 minutes from each time point.

The blood samples were centrifuged at 7,000×g for five minutes in a microcentrifuge (1–15P microfuge, Sigma, Shropshire, UK) to isolate plasma, which was then stored at −21°C until analysis. Plasma corticosterone levels were quantified in duplicate with an enzyme-linked immunosorbent assay (ELISA) (EIA-4164; DRG Diagnostics, Marburg, Germany) in accordance with the manufacturer's instructions.

### Pathology

**Figure 1:**
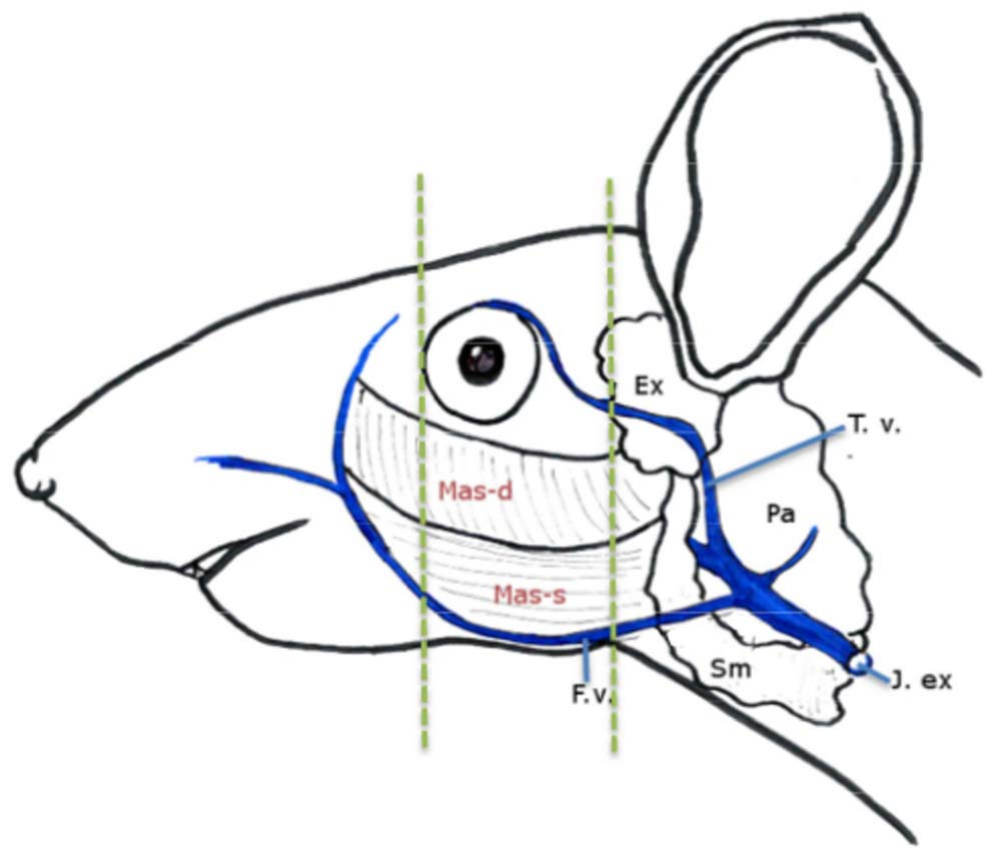
Anatomical overview. Blood samples were taken from either the facial vein (F. v.) or through the medial canthus of the eye to puncture the retro-bulbar venous sinus. Related anatomical structures in head are the extraorbital lacrimal gland (Ex), the parotid gland (Pa), the submandibular gland (Sm), the deep (Mas-d) and superficial masseter muscles (Mas-s), the superficial temporal vein (T.v.) and the external jugular vein (J. ex). The green dashed lines indicate the sections for histopathology. Nomenclature and anatomy are based on Popesko *et al.* [29]

Twenty-four hours after blood sampling, the mice were transferred to a designated necropsy room. The mice were decapitated and the skin covering the skull was removed to inspect for subcutaneous changes. Thereafter, the head was bisected along the midline using a razor blade and immersed in a 4% buffered formaldehyde solution (Gurr formaldehyde, VWR, Vienna, Austria). After three days the tissues were transferred to 70% ethanol (Kemetyl, Køge, Denmark). Before paraffin infiltration, the specimens were immersed for 10 days in a decalcification solution, consisting of a 1∶1 mixture of 44% formic acid (GPR RECTAPUR Myresyre, VWR, Herlev Denmark) and a 20% sodium citrate (Merck Millipore tri-natriumcitrat dihydrat, VWR, Herlev, Denmark) in an aqueous solution. Cross sections of each side of the skull were obtained after decalcification at the level of the medial canthus for eye sections and at the caudal part of the masseter muscle for cheek sections, as indicated in Figure 1, where the rostral sides of the sections were placed face down in the cassettes. Trimmed tissue was embedded in paraffin according to standard procedures [31], 4 µm sections were cut on a rotary microtome (Thermo Scientific HM355S, AX-lab, Vedbæk, Denmark) and stained with hematoxylin and eosin. Three sections, each separated by a 50 µm distance, were evaluated per site. As the control mice had been euthanized in connection to the blood sampling, the cheeks from mice subjected to RSP served as control samples for the mice that had been subjected to FVP and vice versa.

### Statistics

Data were analyzed in SPSS Statistics 20 (IBM, Armonk, NY, USA) and analyzed for normality using Shapiro-Wilk's tests on either non-transformed or log-transformed data. Normally distributed data sets were analyzed with either a one-way analysis of variance (ANOVA) or a multivariate ANOVA for comparing the overall difference within and between groups and with Tukey's *post hoc* tests. Levene's test of equality of sample variances was conducted to test whether the variances were equal across groups. Statistics are presented as an F-value, F(df_w_, df_b_), where df are the degrees of freedom within and between groups, respectively. Body weight and food consumption data were, furthermore, subjected to repeated measures ANOVA. Data sets that did not follow a Gaussian distribution were analyzed with Kruskal-Wallis H-test, where statistics are presented as an H-value, H(df), or a Mann-Whitney U-test, where statistics are present as a *U*-value, as well as the asymptotic significance (2-tailed) *p*-value. P-values <0.05 were considered significant.

## Results

### Body weight and food consumption

**Figure 2:**
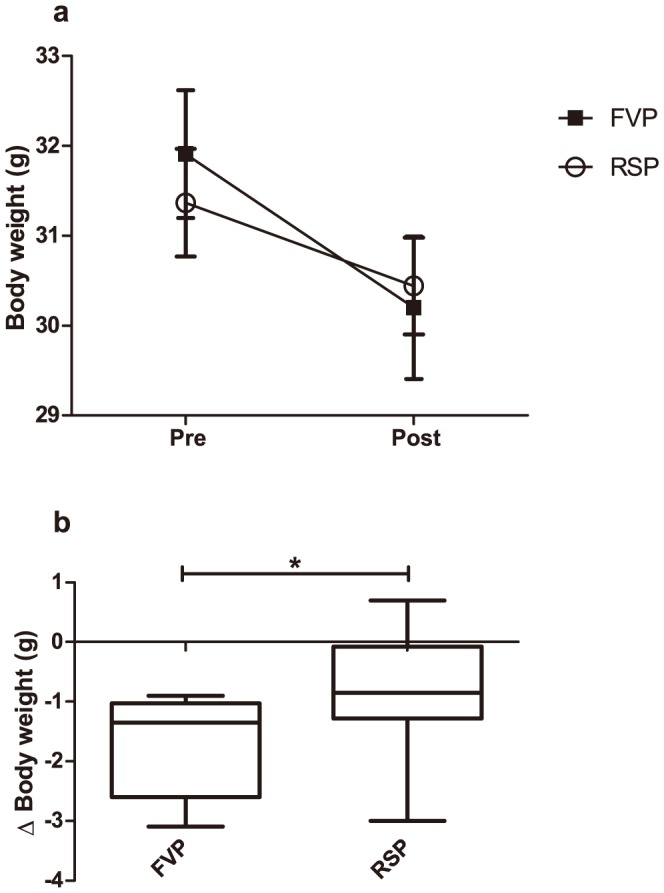
Body weights in mice subjected to facial vein phlebotomy (FVP, N = 12) and retro-bulbar sinus puncture (RSP, N = 12). In 2a, pre-procedural (Day 1) and post-procedural (Day 2) measurements (mean ± SEM) are shown. Control mice (95% CI; 26.5 – 35.8 g) were also weighed at Day 1, but were euthanized in connection to blood sampling as trunk blood was obtained, why body weights were not available at Day 2 and therefore not shown. Mice sampled by FVP (repeated measures ANOVA, p < 0.001) and RSP (repeated measures ANOVA, p = 0.010) lost weight significantly after the blood sampling (2a). In 2b, the body weight loss (ΔBody weight, median with min. to max. values) is shown. The body weight loss was significantly greater in FVP mice than in RSP mice (Mann-Whitney U-test, p = 0.040). The lost blood volume in connection to blood sampling has not been included in the calculation of the body weight loss.

Pre-procedural body weights (Pre) were not significantly different between the three groups (F(2)  = 0.315, *p* = 0.733). Post-procedural body weights (Post) were not significantly different between groups either (F(1)  = 0.064, *p* = 0.803). The repeated measures ANOVA, however, revealed an overall significant decrease in body weights between the first and the second measurement (F(1.000, 22.000)  = 47.695, *p* <0.001), which was also evident, when analyzing the FVP group (F(1.000, 11.000)  = 52.852, *p* <0.001) and RSP group (F(1.000, 11.000)  = 9.489, *p* = 0.010) individually (Figure 2a). The Body weight loss (ΔBW) was significantly higher in FVP mice than in mice subjected to RSP (*U* = 36.500, *p* = 0.040) (Figure 2b). Data are available through Figshare at http://dx.doi.org/10.6084/m9.figshare.1138671.

**Figure 3:**
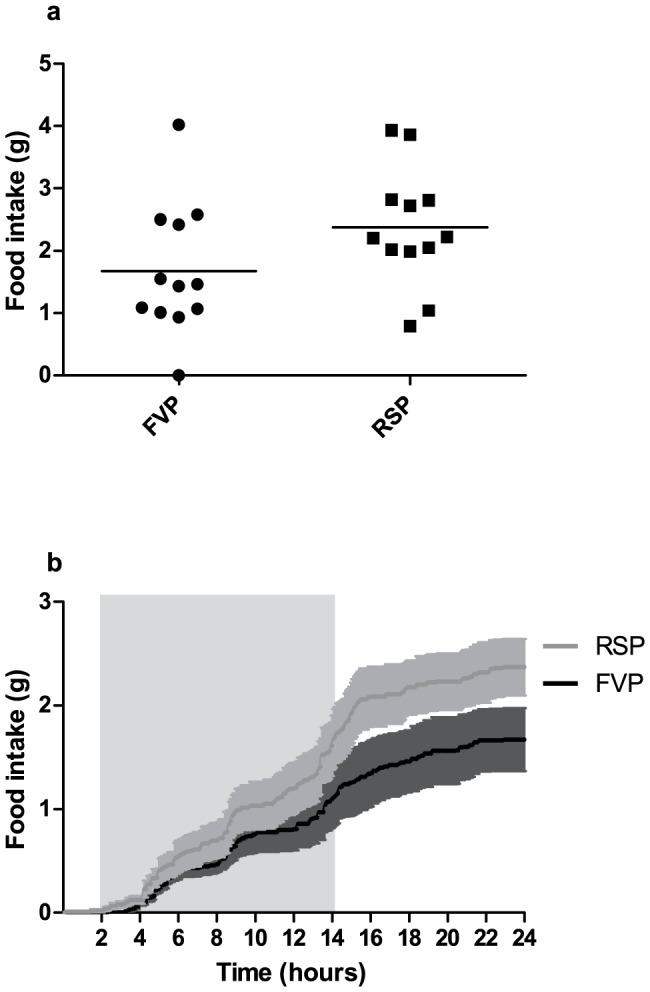
Food consumption during 24 hours after blood sampling in mice sampled by facial vein phlebotomy (FVP, N = 12) and retro-bulbar sinus puncture (RSP, N = 12). In 3a, the total food consumption is shown. The lines indicate means. In 3b, the cumulative food consumption, recorded automatically in a feeding monitoring system during 24 hours, is shown; means (lines) ± SEM (light and dark grey areas). Blood samples were obtained at 4 and 6 pm. The final blood sample was obtained at the beginning of the dark period (grey box). No significant difference between groups was found (ANOVA, *P* = 0.101).

The mean daily food consumption recorded during four days prior to experimentation did not differ significantly between groups. Mice subjected to FVP and RSP consumed significantly less feed during the 24 hours after blood sampling compared to pre-experimental levels. The reduced food consumption, following the blood sampling, did not differ between groups (Figure S1 and Table S1 in File S1). The food consumption was not significantly different between groups at two, four, six, twelve and 24 hours post-procedure (Figure 3a, Table 1), although there was a tendency towards less food consumption in mice subjected to FVP during the 24 hours after blood sampling (Figure 3b) (data are available at http://dx.doi.org/10.6084/m9.figshare.1138672). Neither did the number of meal counts or the meal size, defined as the food intake (gram) per meal, differ between groups at any time point after blood sampling (Table 1). However, a tendency towards smaller meal sizes for FVP mice was observed during the first two hours post-procedure.

**Table 1:** The food consumption (g), number of meal counts and the meal size (g/meal) for mice subjected to facial vein phlebotomy (FVP, N = 12) and retro-bulbar sinus puncture (RSP, N = 12) at two, four, six, twelve and 24 hours (h) after blood sampling (mean ± SEM), analyzed with either a one-way ANOVA (illustrated with a F-value and degrees freedom (F(1))) or a Mann-Whitney U-test (illustrated with a U-value).

Food consumption (g)						
Group		2 h	4 h	6 h	12 h	24 h
	Statistics	U = 60.000,*p* = 0.148	F(1) = 1.488, *p* = 0.235	U = 58.500, *p* = 0.436	U = 49.500, *p* = 0.194	F(1) = 2.927, *p* = 0.101
**FVP**	Mean ± SEM	0.000±0.000	0.095±0.026	0.390±0.067	0.803±0.201	1.672±0.304
**RSP**	Mean ± SEM	0.022±0.015	0.124±0.036	0.535±0.159	1.196±0.248	2.371±0.273
**Meal counts**						
	Statistics	U = 47.500, *p* = 0.061	F(1) = 0.518, *p* = 0.479	U = 60.000, *p* = 0.486	F(1) = 0.303, *p* = 0.588	F(1) = 1.644, *p* = 0.213
**FVP**	Mean ± SEM	0.083±0.083	3.250±0.799	5.583±1.411	9.167±1.669	15.583±1.983
**RSP**	Mean ± SEM	0.500±0.195	2.500±0.669	4.583±1.300	10.750±2.346	20.083±2.896
**Meal size (g/meal)**						
	Statistics	U = 46.500, *p* = 0.053	F(1) = 3.167, *p* = 0.092	F(1) = 2.563, *p* = 0.126	F(1) = 2.132, *p* = 0.159	F(1) = 0.718, *p* = 0.412
**FVP**	Mean ± SEM	0.004±0.004	0.227±0.037	0.433±0.082	0.907±0.248	1.672±0.304
**RSP**	Mean ± SEM	0.069±0.031	0.394±0.121	0.601±0.169	1.300±0.264	2.360±0.275

No difference was found between groups for any parameter at any time point, although a tendency towards smaller meal size was observed for FVP mice the first two hours after blood sampling.

### Plasma corticosterone

**Figure 4:**
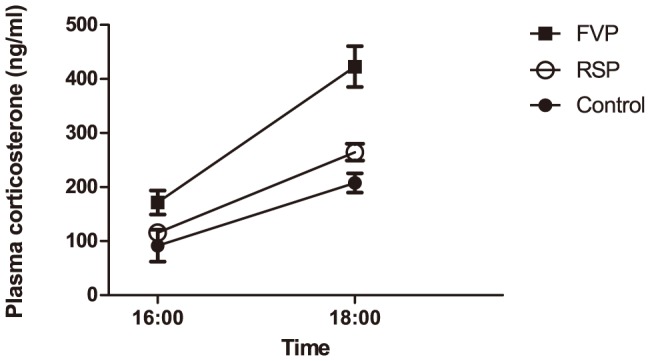
Plasma corticosterone levels in mice subjected to facial vein phlebotomy (FVP, N = 12), retro-bulbar sinus puncture (RSP, N = 12) and decapitation (Control, N = 4 at respective time points). Shown are mean ± SEM. Plasma corticosterone levels of FVP mice were significantly increased at 4 pm compared to the control mice (ANOVA, *p* = 0.046) and at 6 pm compared to RSP mice (ANOVA, *p* = 0.001) and control mice (ANOVA, *p* < 0.001). Notice also the natural increase in circulating corticosterone concentrations as a result of the diurnal rhythm, as illustrated by the control mice.

Log-transformed plasma corticosterone levels (Figure 4) were overall significantly different between groups (F(4,44) = 6.144, *p* = 0.001), where mice subjected to FVP had significantly elevated plasma corticosterone levels at 4 pm compared to the control mice (*p* = 0.046) and at 6 pm compared to mice subjected to RSP (*p* = 0.001) and control mice (*p* <0.001) (data are available at http://dx.doi.org/10.6084/m9.figshare.1138673).

### Pathology

At gross necropsy, visible hematomas were found subcutaneously at the site of blood sampling in both groups of blood sampled mice. As summarized in Table 2, no significant difference was found between the two groups (H(1)  = 0.3260, p = 0.568) for the number of visible subcutaneous hematomas. However, FVP mice had significantly more hematomas in the right cheek than in the left (H(1)  = 7.881, p < 0.01), while no such difference was found in the RSP mice (H(1)  = 0.161, p = 0.688).

**Table 2:** Macroscopic subcutaneous hematomas.

Sub-cutaneous hematomas	Facial vein phlebotomy	Retro-bulbar sinus puncture
Right side	9	7
Left side	2	6

The table shows the number of mice, which were subjected to facial vein phlebotomy (FVP, N = 12) and retro-bulbar sinus puncture (RSP, N = 12), with visible sub-cutaneous hematomas at the site of blood sampling, as scored at gross necropsy. No overall difference was found between groups for the number of visible subcutaneous hematomas (Kruskal-Wallis H-test, p = 0.568). However, FVP mice had significantly more hematomas in the right cheek than in the left (Kruskal-Wallis H-test, p <0.01). No difference was found between the sides for RSP mice (Kruskal-Wallis H-test, p = 0.688).

**Figure 5:**
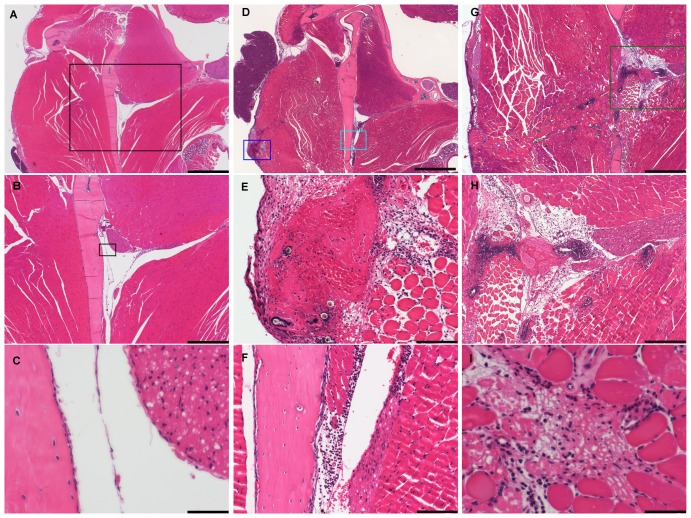
Pathological changes in the cheeks and associated tissue. Shown are examples of control sections (A-C) and sections from mice, which were subjected to facial vein phlebotomy (D-I). A) An overview is given, showing the temporo-mandibular joint, the mandible and masseter muscle. The area highlighted by the black box is shown in higher magnifications in B and C, respectively. D) Overview. E) The area highlighted by the dark blue box in D is shown in higher magnification, illustrating the puncture site after facial vein phlebotomy. Note the extensive hemorrhage with a central blood clot, deposited hair and diffuse polymorphonuclear cell infiltration. F) The area highlighted by the light blue box in D is shown in higher magnification. Note diffuse inflammation of the muscle, which involves the medial side of the mandible and the caudal end of the associated nerve (tentatively the lingual nerve). G) The puncture site after facial vein phlebotomy in another mouse, showing the puncture tract, created by the lancet, as evidenced by inflammatory cells and hair in the masseter muscle. H) A higher magnification of the area highlighted by the green box in G. Note the thrombus inside the vessel (possibly the facial vein), hair deposition and extensive cell infiltration. I) Muscle cell necrosis. All sections were stained with hematoxylin and eosin. Bars = 1000 µm in A and D. Bars = 500 µm in B and G. Bars = 100 µm in E, F and H. Bars = 50 µm in C and I.

Histopathological evaluations revealed acute inflammatory changes in all animals at the site of venipuncture and in surrounding tissues (Table 3). In the cheeks (Figure 5; A-I) of FVP mice (N = 12), acute inflammation, dominated by infiltration of polymorphonuclear (PMN) cells was demonstrated in subcutaneous muscle fascia as well as the masseter muscle. In the left cheek of eight mice and in the right cheek of seven mice, inflammatory foci surrounding hair, which had likely been introduced with the lancet, and a central core of cellular debris were demonstrated (Figure 5; E, G, H). Perineuritis and inflammation of the mandible periost, primarily located to the medial side of the mandible, could also be demonstrated in some mice (Figure 5; D, F). In one case, PMNs were found in the leptomininges (occipital lobe).

**Table 3:** Histopathological changes in mice subjected to facial vein phlebotomy and retro-bulbar sinus puncture.

Organ/structure	Facial vein phlebotomy(N = 12)				Retro-bulbar sinus puncture(N = 12)			
Left cheek	I	N	H	Hair	I	N	H	Hair
*Subcutaneous muscle fascia*	12/12	10/12	12/12	8/12	0/12	0/12	0/12	0/12
*Masseter muscle*	12/12	8/12	7/12	4/12	0/12	0/12	0/12	0/12
*Mandible periost*	7/12	0/12	0/12	0/12	0/12	0/12	0/12	0/12
*Perineurium of facial nerves*	1/12	0/12	0/12	0/12	0/12	0/12	0/12	0/12
Right cheek								
*Subcutaneous muscle fascia*	12/12	6/12	12/12	6/12	0/12	0/12	0/12	0/12
*Masseter muscle*	12/12	8/12	10/12	1/12	0/12	0/12	0/12	0/12
*Mandible periost*	8/12	0/12	4/12	0/12	0/12	0/12	0/12	0/12
*Perineurium of facial nerves*	3/12	0/12	0/12	0/12	0/12	0/12	0/12	0/12
Left eye								
Palpebra*	0/10	0/10	0/10	0/10	4/11	2/11	5/11	0/11
Nictitating membrane*	0/11	0/11	0/11	0/11	11/11	11/11	11/11	0/11
*Cornea*	0/12	0/12	0/12	0/12	7/12	2/12	5/12	0/12
*Sclera*	0/12	0/12	0/12	0/12	8/12	6/12	6/12	0/12
*Harderian gland*	0/12	0/12	0/12	0/12	11/12	10/12	10/12	0/12
*Retrobulbar connective tissue and orbital muscles*	0/12	0/12	0/12	0/12	12/12	11/12	12/12	4/12
Right eye								
Palpebra*	0/11	0/11	0/11	0/11	3/9	1/9	2/9	0/9
Nictitating membrane*	0/11	0/11	0/11	0/11	9/9	8/9	8/9	1/9
Cornea*	0/11	0/11	0/11	0/11	5/12	3/12	4/12	0/12
Sclera*	0/11	0/11	0/11	0/11	8/12	8/12	6/12	0/12
Harderian gland*	1/11	1/11	1/11	1/11	9/12	8/12	8/12	0/12
Retrobulbar connective tissue and orbital muscles*	0/11	0/11	0/11	0/11	12/12	12/12	12/12	4/12

The number of mice, showing histopathological changes in the evaluated structures is given. Changes involved acute inflammation, dominated by the infiltration of polymorphonuclear cells (I), necrosis (N) and hemorrhage (H). In some cases, the presence of hair was noted. Note that some animals may have been noted for changes in several structures. *This structure was unavailable for evaluation in all mice.

**Figure 6:**
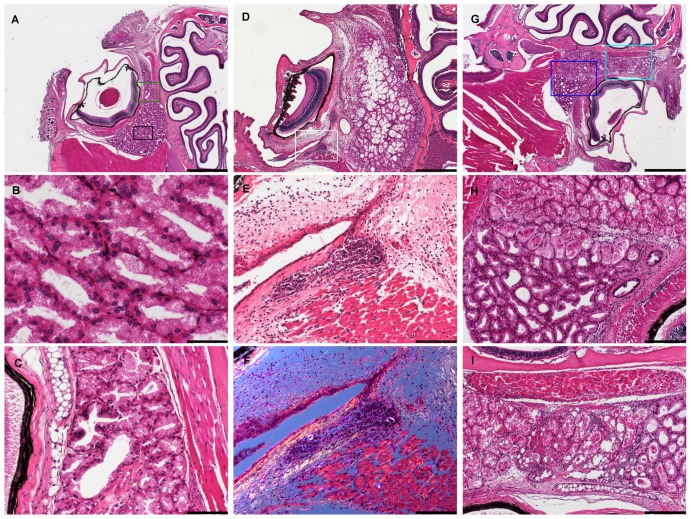
Pathological changes in orbits and associated structures. Shown are examples of control sections (A-C) and sections from mice, which were subjected to retro–bulbar sinus puncture (D-I). A) An overview is given, showing the palpebrae, eye and the Harderian gland as well as the ethmoid turbinates of the nasal passage. The cornea is artefactually folded. B) A higher magnification of the area in the black box in A. Shown is normal histology of the mouse Harderian gland. C) Higher magnification of the area lined by the green box in A, showing the caudal part of the retina photoreceptor segment and the pigmented epithelium, the nictitating membrane, Harderian gland and extraorbital muscle. D) Overview. Note the corneal thickening due to inflammation, retrobulbar inflammation and edema as well as Harderian gland necrosis. Note also the inflammatory foci, highlighted in the white box, which surround embedded hair. E) Higher magnification of the area in the white box in D, showing diffuse edema and inflammation in the retro-bulbar connective tissue as well as the inflammatory foci with embedded hair. F) The same section as in E with polarized light for identification of keratin from the embedded hair. G) Overview. H) The area marked by the blue box in G is shown in higher magnification. Note the partial necrosis of the Harderian gland, delineated by a clear demarcation line, which suggests ischemic necrosis. I) Higher magnification of the area marked by the light blue box in G, showing retro-bulbar inflammation of both the Harderian gland and extraorbital muscle and necrosis of the Harderian gland. All sections were stained with hematoxylin and eosin. Bars = 1000 µm in A and G. Bar = 500 µm in D. Bars = 200 µm in H and I. Bars = 100 µm in C, E and F. Bar = 50 µm in B.

In mice sampled from the retro-orbital sinus (N = 12), retro-orbital PMN cell infiltration was identified in all cases (Figure 6; A-1) and in seven mice the acute inflammatory reaction, accompanied by oedema and hemorrhage, extended from the retro-bulbar connective tissue and orbital muscles around the eye through the sclera to also involving the cornea (Figure 6D). These changes extended in all cases into the nictitating membrane and in five mice also the palpebrae, in one mouse these structures could not be evaluated, as they had not been included in the section. Abrasion of the sclera with concurrent ulceration was seen in two mice (Figure 6D). The Harderian gland was in 11 cases on the left side and eight cases on the right side infiltrated with inflammatory cells. The gland was in all of these cases showing changes from mild degeneration and ectasia of acini to severe necrosis of major parts of the gland (Figure 6; G-I). On corresponding control sections from the mice in the FVP group, inflammation and necrosis of the Harderian gland could also be demonstrated in one mouse. Otherwise, none of the above described lesions could be identified on the control sections. The sections of the right eye from one control mouse had, however, not been handled properly and could not be evaluated. As with facial vein phlebotomy, inflammatory foci, encircling embedded hair that had likely been introduced with the capillary tube, were in 5 cases observed in the left orbit and in 4 cases in the right orbital tissues (Figure 6; E-F).

## Discussion

A number of studies have investigated the welfare implications of RSP in rats [8], [10], [14], [32] and compared this technique with other blood sampling methods [6], [9], [13]. However, only a limited number of publications describe the impacts of retro-bulbar blood sampling in mice [12], [33] and only one study compares this method to venipuncture of the facial vein (sometimes incorrectly referred to as the submandibular vein) [34]. Occasionally, the superficial temporal vein is used rather than the facial vein. However, in our opinion this is not recommended due to the close proximity of the inner ear (Figure 1) and thus the risk of injuring very sensitive structures.

The two experimenters performing the blood samplings had extensive experience in one of the techniques respectively and thus were responsible for blood sampling either by FVP or RSP. This procedure was chosen to ensure that only experienced and qualified personnel took the respective blood samplings, thus minimizing any trauma related to inaccurately performed techniques.

Body weight and food consumption are well validated sensitive markers of animal welfare [1], [35] and loss of body weight and reduced food intake have been demonstrated after stressful procedures in mice in several studies [36]–[39]. In the present study, all mice that were sampled for blood lost weight significantly, and the body weight loss was significantly greater in mice subjected to FVP than in mice subjected to RSP. Both groups also consumed less feed following the blood sampling compared to pre-experimental levels. Although, no difference was found for the food consumption or in the feeding pattern between mice sampled by FVP and RSP after the blood sampling, a trend towards lower food consumption and smaller meal size was observed in the mice subjected to FVP.

A number of studies have shown that blood sampling procedures, depending on several factors like the method used as well as experience and skill of the experimenter, will affect circulating stress hormone levels through an activation of the hypothalamic-pituitary-adrenal (HPA) axis [33], [40]–[42] and the sympathetic nervous system [4]. Therefore, for animal welfare reasons and to ensure minimal confounding influence on the scientific result it is important that the blood sampling method is minimally stressful. Corticosterone, being the major effector hormone of the HPA axis in rodents [43]–[45], is widely used as a measure of stress [46]–[48]. Normally, circulating concentrations of corticosterone follow a diurnal rhythm that in mice peaks in late afternoon at levels between 150–200 ng/ml and then declines during the night [43], [47]. Thus, plasma corticosterone levels increased in all groups from 4 to 6 pm as expected. However, this increase was significantly higher in mice subjected to FVP than in both control mice and mice subjected to RSP, and plasma corticosterone levels were significantly elevated in mice subjected to FVP at both time points. Thus, sampling blood from the cheeks of the mice may stimulate glucocorticoid secretion to a greater extent than from the eye. As all mice, despite group, were restrained in the same manner in relation to blood sampling, the stress response associated with the restraint cannot explain the difference in plasma corticosterone concentrations between groups. It is possible that venipuncture of the facial vein may result in a higher pain response than RSP, as the masseter muscle, the major masticatory muscle in rodents, is evidently injured by blood sampling. This might explain also the greater BW loss in mice subjected to FVP than in mice subjected to RSP, but no difference in the food consumption or the feeding pattern was found between the groups. On the other hand, corticosterone is also secreted in response to blood loss [49], [50]. Puncturing the facial vein often results in extensive hemorrhage, which can be difficult to control. In some FVP mice, the bleeding continued for a few seconds after releasing the stasis (restraint). No bleeding was observed after RSP.

To assess the degree of continued bleeding, the mice were macroscopically evaluated for subcutaneous hematomas. Both mice sampled by FVP and RSP had macroscopically visible hematomas in the cheeks and around the orbit, respectively. Collecting blood is invasive and will inevitably cause hemorrhage and trauma at the site of blood sampling. Therefore, some degree of hemorrhage was expected in the puncture tract and adjacent tissues, which has also been identified in rats after RSP [10]. Nevertheless, mice subjected to FVP had more hematomas subcutaneously in the right cheek than in the left, indicating that the hematomas may be induced differently between the two sides due to a difference in how the two sides were approached in relation to the angle of the mouse, when fixated. No difference was found between the sides macroscopically for RSP mice and no overall difference in the number of subcutaneous hematomas, counting the sides separately, was found between the two groups. Thus, RSP as well as FVP cause visible hemorrhage in the orbits and cheeks, respectively. In mice subjected to FVP, the hematomas were diffusely located in the subcutis. In mice subjected to RSP, the hemorrhage had extended from the puncture tract and was located around the orbit post-mortem. Although not quantitatively evaluated, the hemorrhage appeared to involve a larger area for FVP mice.

Acute inflammatory lesions were expected in both groups of blood sampled mice as a response to the local tissue damage, caused by the punctures. However, the extent of tissue damage observed in both experimental groups may be indicative of welfare impairment. A consistent finding in both groups was the deposition of hairs along the canal in the tissue produced by the lancet or capillary tube, causing focal inflammatory reactions. As these sections were obtained at 24 hours post-sampling, only acute inflammatory reactions were demonstrated. However, hair acts as a foreign material when deposited in tissues, characteristically giving rise to a chronic foreign body reaction [51], [52].

FVP resulted in severe acute inflammation, dominated by infiltration of PMN cells in the subcutaneous muscle fascia as well as the masseter muscles. As the skin including the subcutis had been removed at gross necropsy, these structures could not be evaluated. The cell infiltration extended deep into the muscle tissue, and in many cases the puncture tract created by the lancet could be identified by the infiltration of inflammatory cells and deposited hair (Figure 6G). Furthermore, in several mice, the inflammation extended to the medial side of the mandible, also involving the periost. Perineuritis of the nerve in close approximation to the medial side of the mandible, tentatively the lingual nerve, was another frequent finding.

In one case, a local acute inflammatory meningeal reaction was identified and on orbital control sections from this mouse Harderian gland necrosis could also be demonstrated. This suggests that hematogenously carried materials had settled in minor vessels supplying associated tissues. It is possible that epidermal bacteria were introduced by the lancet and transferred to the blood during the venipuncture. Another possible explanation is the circulation of microthrombi, created at the puncture site, which then settled in smaller vessels, causing ischemic necrosis of approximating structures, such as the Harderian gland. Thrombosis of a major vessel at the puncture site, possibly the facial vein, could be identified on some sections (Figure 5H). This raises some concern with using the FVP technique on immunocompromised mice.

RSP elicited equally major tissue trauma to the orbital and associated tissues. Inflammation in the orbit, occasionally extending around the whole eye, was demonstrated in several mice. In some cases corneal inflammation was accompanied by edema and in two cases abrasion of the sclera, likely caused by entrance of the capillary tube, could be demonstrated. Ocular inflammation and trauma may subsequently lead to degeneration, neovascularization and blindness [53].

The Harderian gland was also affected by the blood sampling. This gland produces lipids and porphyrin and the main function is, together with the intraorbital lacrimal gland, to lubricate the orbit, nictitating membrane and cornea [54]. Furthermore, it may also serve a function in maintaining the tear film [53]. Since the Harderian gland is located in close approximation to the venous sinus, it is at risk of traumatization [54]. Whether the necrosis was traumatically caused by the capillary tube or due to ischemic changes after laceration of the blood supply, or both, is unclear. A sharp demarcation line (Figure 5H) could be demonstrated in some cases between normal and necrotic tissue in the Harderian gland, which may suggest that the necrosis was at least partially produced by ischemia.

It is unknown whether loss of function of the Harderian gland has a pathophysiological impact, since orbital lubrication is facilitated also by the lacrimal gland. However, Harderian gland necrosis may likely cause significant chronic alterations [12]. As RSP in mice is most often a survival procedure, it may potentially influence animal health later on. Therefore, evaluation of the Harderian gland necrosis during a longer time perspective after retro-bulbar blood sampling needs to be further studied. Other possible complications like disturbances in the orbital blood supply or chronic inflammatory reactions, e.g. due to foreign body reactions to hair, are equally important to investigate. Blindness and endophthalmia has been reported in rats after RSP [7], [8]. Mice, being smaller than rats, may respond with more severe tissue trauma and may be more affected than rats subjected to similar procedures. Thus, more studies on the chronicity of tissue changes after retro-bulbar blood sampling in mice are needed.

In conclusion, facial vein phlebotomy seems to induce a higher stress response and a greater body weight loss than retro-bulbar blood sampling in mice. However, pathological evaluations revealed extensive tissue damage after both facial vein phlebotomy and retro-orbital puncture. This study demonstrates that routine blood sampling has a considerable impact on animal welfare, which should be considered whenever blood samples are obtained. Continuous refinement of blood sampling methods is important to improve animal welfare and scientific quality.

## Supporting Information

File S1 **Additional information on the daily food consumption including pre-sampling levels.**
(DOCX)
