# Supplementary material for: Physiological and Pathological Impact of Blood Sampling by Retro-Bulbar Sinus Puncture and Facial Vein Phlebotomy in Laboratory Mice
Source: PLoS One. 2014 Nov 26;9(11):e113225. doi: 10.1371/journal.pone.0113225 (PMC4245142; doi:10.1371/journal.pone.0113225)

File S1. Additional information on the daily food consumption including pre-sampling levels.

Table S1. Food consumption before and after blood sampling. The table shows the daily (mean ± SD gram) food consumption of male C57BL/6J mice from four days prior to facial vein phlebotomy (FVP, N = 12), retro-bulbar sinus puncture (RSP, N = 12) and decapitation (Control, N = 8) and 24 hours after blood sampling (+1 day). Furthermore, the mean food consumption of the four pre-experimental days is given for each group (baseline). Baseline food consumption did not differ significantly between groups (one-way ANOVA, F(2) = 1.113, *p* = 0.342). Mice subjected to FVP consumed significant less feed during the 24 hours after blood sampling compared baseline levels (repeated measures ANOVA, F(1.000, 11.000) = 55.899, p < 0.001), as did mice subjected RSP (repeated measures ANOVA, (F(1.000, 11.000) = 15.281, *p* = 0.002). This decrease in food consumption did not differ between FVP mice and RSP mice (one-way ANOVA, F(1) = 2.384, p = 0.137). As the control animals were euthanized in connection to blood sampling, the food consumption post-sampling could not be recorded for these animals (-).

|  | Days in relation to blood sampling | | | | | |
| --- | --- | --- | --- | --- | --- | --- |
| Group | -4 | -3 | -2 | -1 | Baseline | +1 |
| FVP (N = 12) | 4,199 ± 1.190 | 3.985 ± 0.713 | 3.810 ± 1.215 | 3.681 ± 0.722 | 3.919 ± 0.978 | 1.672 ± 0.304 |
| RSP (N = 12) | 3.865 ± 1.117 | 3.768 ± 1.117 | 4.112 ± 0.855 | 3.722 ± 0.812 | 3.867 ± 0.881 | 2.371 ± 0.273 |
| Control (N = 8) | 4.316 ± 1.111 | 4.571 ± 0.770 | 4.407 ± 0.943 | 3.729 ± 0.740 | 4.256 ± 0.917 | - |

Figure S1. Daily food consumption. Shown is the daily food intake in g (mean ± SEM) from four days prior to blood sampling and at Day 1 (24 hours after blood sampling). The food consumption decreased in both mice subjected to facial vein phlebotomy (FVP, **p-value < 0.001) and retro-bulbar sinus puncture (RSP, *p-value < 0.005) compared to mean pre-experimental levels. The reduction in food consumption following the blood sampling did not differ between groups.


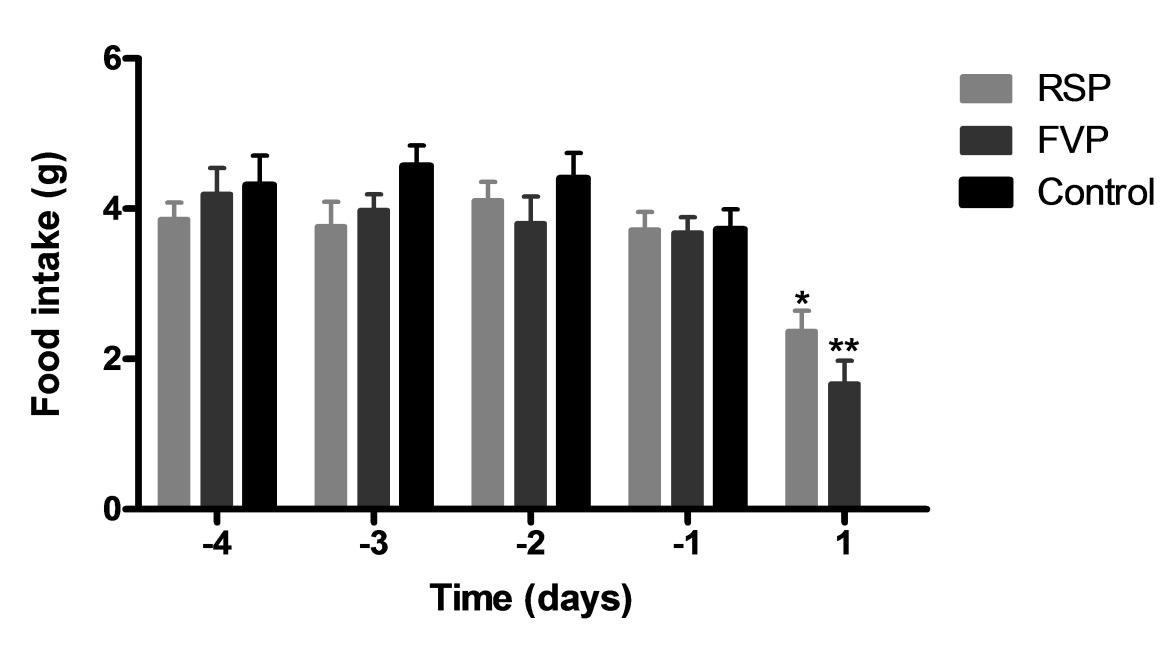

Supplement: File S1 — Additional information on the daily food consumption including pre-sampling levels. (DOCX) [file pone.0113225.s001.docx]
